# Clinical Significance of Maximum Intensity Projection Method for Diagnostic Imaging of Thoracic Outlet Syndrome

**DOI:** 10.3390/diagnostics13020319

**Published:** 2023-01-15

**Authors:** Takeshi Ogawa, Shinzo Onishi, Naotaka Mamizuka, Yuichi Yoshii, Kazuhiro Ikeda, Takeo Mammoto, Masashi Yamazaki

**Affiliations:** 1 Department of Orthopedic Surgery, National Hospital Organization Mito Medical Center, 280 Sakuranosato, Ibarakimachi 311-3193, Japan; 2 Department of Orthopedic Surgery and Sports Medicine, Mito Clinical Education and Training Center, University of Tsukuba Hospital, Mito Kyodo General Hospital, 3-2-7 Miya-Machi, Mito 310-0015, Japan; 3 Department of Orthopedic Surgery, Faculty of Medicine, University of Tsukuba, Tsukuba 305-8577, Japan; 4 Baseball and Sports Clinic, 2-228-1 Kosugi, Park City Musashikosugi the Garden Towers West 1st Floor W4, Nakahara-Ward, Kawasaki 211-0063, Japan; 5 Department of Orthopedic Surgery, Tokyo Medical University Ibaraki Medical Center, Ami 300-0395, Japan; 6 Department of Orthopedic Surgery, Kikkoman General Hospital, Noda 278-0005, Japan

**Keywords:** maximum intensity projection, subclavian vein, thoracic outlet syndrome

## Abstract

The aim of this study was to use the magnetic resonance imaging maximum-intensity projection (MRI-MIP) method for diagnostic imaging of thoracic outlet syndrome (TOS) and to investigate the stricture ratios of the subclavian artery (SCA), subclavian vein (SCV), and brachial plexus bundle (BP). A total of 113 patients with clinically suspected TOS were evaluated. MRI was performed in a position similar to the Wright test. The stricture was classified into four grades. Then, the stricture ratios of the SCA, SCV, and BP in the sagittal view were calculated by dividing the minimum diameter by the maximum diameter of each structure. Patients were divided into two groups: surgical (*n* = 22) and conservative (*n* = 91). Statistical analysis was performed using the Mann–Whitney U test. The stricture level and ratio in the SCV were significantly higher in the surgical group, while the stricture level and the ratio of SCA to BP did not show significant differences between the two groups. The MRI-MIP method may be helpful for both subsidiary and severe diagnoses of TOS.

## 1. Introduction

**Figure 1:**
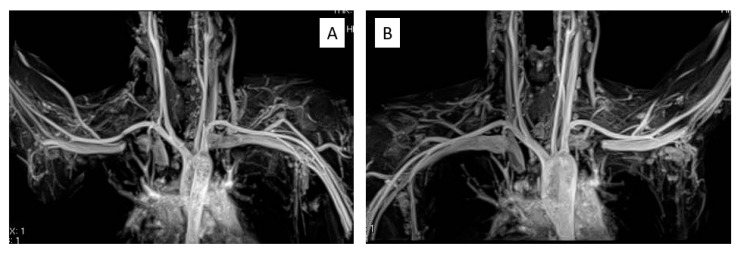
MRI-MIP display from the chest to the upper arm. (**A**) Raised right arm. (**B**) Raised left arm. MRI-MIP, magnetic resonance imaging maximum-intensity projection.

Thoracic outlet syndrome (TOS) is probably more common than is believed, especially among young people. Diagnosing TOS is difficult because no reliable mechanical examination has been shown [1,2,3,4,5,6,7]. However, TOS has a wide variety of symptoms, and its pathogenesis is still debated. The diagnosis of TOS is also dependent on various evoked tests, and a classification of disputed neurogenic TOS has been proposed [8,9,10,11,12]. Patients with no abnormal findings on electrophysiological examination but with a variety of subjective symptoms are difficult to diagnose, and cases suspected to have TOS may actually be diagnosed with hysterical paralysis. One possible method, although not very reliable, to diagnose disputed neurogenic TOS is a medial antebrachial cutaneous nerve conduction study [13,14]. Imaging tests such as 3D-CT angiography, 3D-MR angiography, brachial plexus angiography, and angiography are used to visualize stenotic areas in blood vessels and nerves [15,16,17,18,19,20]; however, these cause problems related to radiation exposure and the use of contrast media. In this study, we report the application of the maximum intensity projection (MIP) method in MRI, which is well known for the depiction of the cerebral vasculature, and its use in the diagnosis of TOS. MIP is used to represent the highest intensity values along one axis of a three-dimensional volume in a two-dimensional (2D) image, allowing rapid interpretation of the entire volume based on this 2D projection (Figure 1A,B). The objective of this study was to evaluate the diagnostic significance of the MIP method for TOS. We also classified the degree of stenosis in the subclavian artery (SCA), subclavian vein (SCV), and brachial plexus and calculated the stricture rate to evaluate the usefulness of MRI-MIP images in the diagnosis of TOS.

## 2. Materials and Methods

This study included 113 patients (60 men and 53 women) with clinically suspected TOS who underwent MRI-MIP at our hospital from May 2014 to March 2021, consecutively. The patients had an average age of 32.5 (14–67) yr. The institutional review board of the University of Tsukuba Hospital approved this study (Study Number: NO 22-44). Subjective symptoms varied from pain, numbness, and lethargy in the upper limbs and fingers to tenderness in the oblique interosseous muscle, intercostal space, or pectoralis minor tendon area. Patients with positive Roos test, Wright test, Adson test, or Eden test results were included in this study.

**Figure 2:**
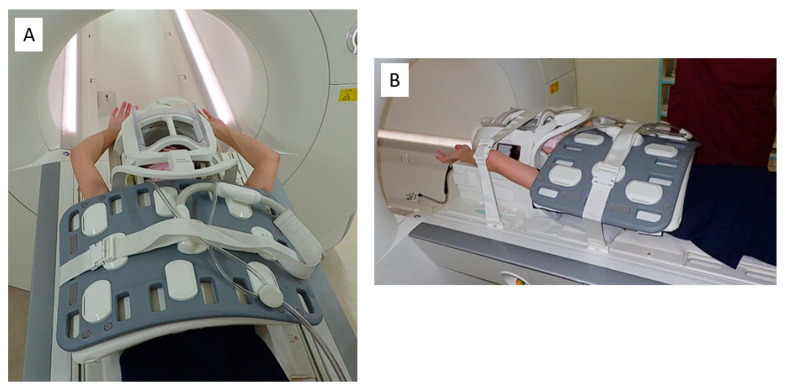
MRI imaging limb position. (**A**) The patient is placed in a supine position with the upper limb further elevated than in the Wright test position. (**B**) The upper extremities from the trunk are secured. MRI, magnetic resonance imaging.

MRI was performed using a clinical 3 Tesla machine (Magneton Skyra 3T, Siemens, Berlin, Germany). The patients were placed in a supine position with the upper limb raised further than that in the Wright test position (Figure 2A). Imaging was performed with the upper limb and trunk firmly fixed with a whole-body coil and bandages (Figure 2B). The imaging conditions were as follows: 3D-STIR with a slice thickness of 1.3 m; FOV, 380 mm; TR/TE, 387/50; matrix, 320 × 256; and flip angle, 120°. A special pulse monitor was attached to the healthy index finger to synchronize imaging with the heartbeat. The subclavian arteries (SCA) and subclavian veins (SCV) were reconstructed using MIP and evaluated in the intercostal space.

**Figure 3:**
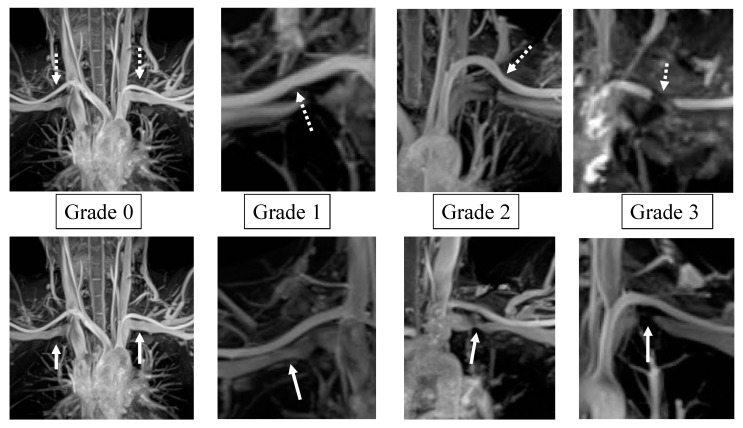
The degree of stenosis of SCA and SCV is classified into 4 levels. Upper row (dotted arrow), a stenosis part of SCA. Lower row (solid line arrow), a stenosis part of SCV. Grade 0; no stenosis. Grade 1; mild stenosis, less than 50%. Grade 2; moderate stenosis, more than 50%. Grade 3; severe stenosis to the point of discontinuity. SCA, subclavian artery; SCV, subclavian vein.

**Figure 4:**
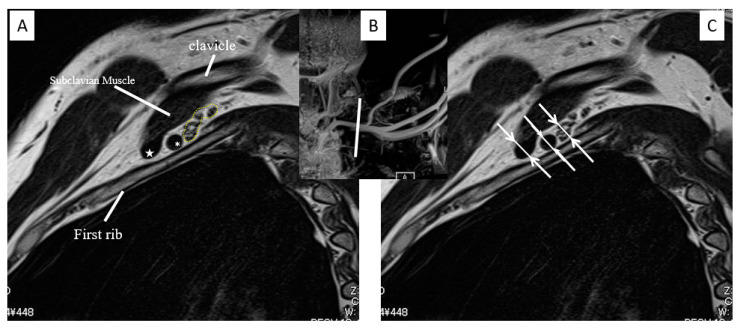
Left sagittal view. (**A**) The SCV (star), SCA (asterisk), and nerve bundle (dot line) are shown. (**B**) Scout view of MRI-MIP image. (**C**) SCA, SCV, and nerve bundle diameter measurements (arrows). MRI-MIP, magnetic resonance imaging maximum-intensity projection; SCA, subclavian artery; SCV, subclavian vein.

The degree of SCA and SCV stenosis in MRI-MIP images were classified into four grades: grade 0 = no stenosis; grade 1 = stenosis < 50% of the maximum diameter of the SCA or SCV; grade 2 = stenosis > 50% of the maximum diameter of the SCA or SCV; and grade 3 = stenosis to the point of interruption (Figure 3). On the second evaluation, we used proton-density-weighted (PDW) sagittal images to quantitatively evaluate the stricture rate of the SCA, SCV, and nerve bundle at the costovertebral gap. The stricture rate was calculated by dividing the minimum diameter (a) of each SCA, SCV, and nerve bundle in the PDW sagittal image by the maximum diameter (b) as follows: (1 − a/b) × 100 (Figure 4). A slice in which the first rib was the longest in a plate-like shape was used as the reference position. The maximum and minimum diameters were measured in the area where the first rib was located. The stricture grade and rate of SCA, SCV, and nerve bundle were statistically compared.

A total of 22 patients underwent surgery (first rib resection), and 91 successfully underwent conservative treatment. The Mann–Whitney U test was used for statistical analysis, and a *p*-value of less than 0.05 was considered significant. Indications for surgery were defined as patients who were clinically diagnosed with TOS, refractory to conservative treatment such as oral medication and rehabilitation, or who requested surgery. In addition, only some cases from both groups were compared in terms of the stenosis rate between the affected and normal sides. None of the patients had symptoms on the healthy side, and only those cases for which data were available were included in this study.

## 3. Results

A total of 22 patients (13 men and 9 women) belonged to the surgical group, with a mean age of 34.8 yr (14–56 yr), and 91 patients (47 men and 44 women) in the conservative group, with a mean age of 33.0 yr (15–67 yr). No significant differences were reported in the demographic profile between the two groups. The main indications for surgery were sports such as baseball in six cases, numbness and pain in the upper limbs that persisted for many years in twelve cases, and a strong cold sensation in addition to numbness in the hands in four cases. The background characteristics of the patients in the surgical group are shown in Table 1. The average duration of illness was 4.6 yr (2–15 yr). A total of three cases were arterial TOS, while the other cases involved neurogenic TOS. Patient satisfaction after surgery at the final follow-up was excellent in seven cases, good in thirteen cases, fair in two cases, and no case was classified as unsatisfactory.

Table 2 shows the breakdown of SCA and SCV stricture grades in MRI-MIP images. The SCV showed a significantly stronger stricture in the surgery group (*p* < 0.01), whereas the SCA showed more grade 0 patients without stricture in both groups, showing no difference between the two groups (*p* = 0.33).

The mean stricture rates of SCA, SCV, and nerve bundle were 34.6%, 76.7%, and 34.5% in the surgical group, and 28%, 67.7%, and 34.5% in the conservative group. There was a significantly higher stricture rate in the surgery group for SCV (*p* = 0.036). In contrast, the SCA (*p* = 0.21) and nerve bundle stenosis rates (*p* = 0.53) did not differ between the two groups (Table 3).

In a comparative study of the affected and normal sides, there were seven and 24 cases in the surgical and conservative groups, respectively (Table 4). A trend was reported towards greater strictures on the affected side of the SCA in the surgery group (*p* = 0.064). However, no significant differences were found in other endpoints between the affected and normal sides.

### Representative Case

**Figure 5:**
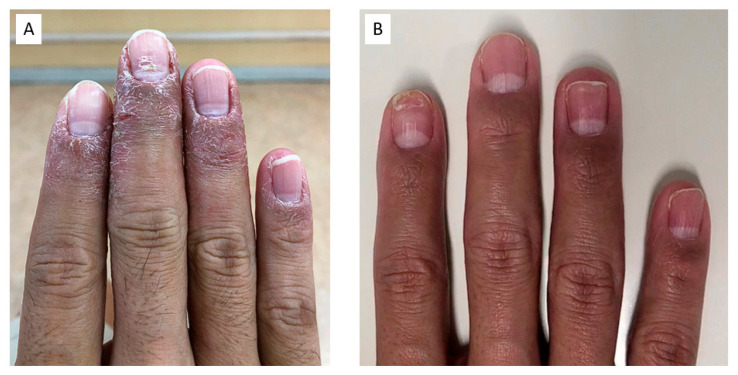
Skin manifestations of the right fingers: (**A**) preoperative finding; (**B**) postoperative finding.

**Figure 6:**
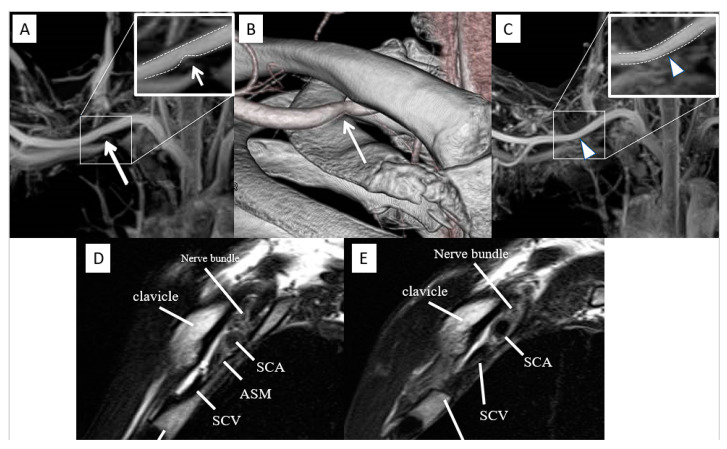
Preoperative and postoperative images. (**A**) Preoperative MRI-MIP image showing grade 1 stenosis of the SCA (arrow). (**B**) Preoperative 3D-CT angiography showing stenosis from below at the site where the SCA meets the clavicle (arrow). (**C**) Postoperative MRI-MIP image revealing stenosis of SCA disappeared and identified as grade 0 (arrowhead). (**D**) Preoperative sagittal view shows that the anterior scalene muscle is in contact with the SCA, and the SCV is disrupted. (**E**) Preoperative sagittal view shows that the first rib was resected, and the anterior scalene muscle, which was in contact with the SCA, was absent. Stenosis of the SCV remained. MRI-MIP, magnetic resonance imaging maximum-intensity projection; SCA, subclavian artery; SCV, subclavian vein.

A 40-year-old man presented with weakness in the right upper limb. He has been experiencing lethargy of the right upper limb for five years, which was aggravated by running. He worked as a researcher, and his symptoms worsened three months ago, making it difficult for him to handle a pipette. He was diagnosed with right TOS by his local doctor and referred to our hospital. He had numbness in the right fingertips, epidermal avulsion of the fingertips, and nail deformity (Figure 5A). The Roos and Wright test results were positive. He was limited to 20 s in the Roos test due to numbness and sluggishness in his right arm, and his hand turned pale. Radiography showed no cervical ribs, while MRI-MIP image and 3D-CT angiography showed stenosis of the SCA (grade 1) and SCV (grade 3) in the intercostal space (Figure 6A,B,D). The patient opted to undergo surgery; hence, first rib resection using the transaxial approach [7] was performed. Postoperatively, numbness in the right upper extremity decreased, and skin lesions on the tips of the fingers improved after three months (Figure 5B). Limping of the upper extremities during running also disappeared. One year after surgery, the MRI-MIP images showed no stenosis of the SCA (grade 0), and the sagittal section showed that the anterior scalene muscle, which was in contact with the SCA before surgery, had disappeared (Figure 6C,E).

## 4. Discussion

In this study, MRI-MIP imaging showed that the stricture rate of the SCV reflected the severity of TOS, whereas the stricture of the SCA and nerve bundles was independent of severity, despite the absence of venous TOS. The application of MRI-MIP imaging in the diagnosis of TOS has been reported in only one case by Esposito et al. [21]. Zhang et al. described the usefulness of contrast-enhanced magnetic resonance angiography for the diagnosis of TOS in 27 cases [22]. Hardy et al. investigated the accuracy of MRI diagnosis in 48 TOS cases and reported its usefulness [23]. This study evaluated 113 MRI-MIP images, making it the largest survey conducted to date.

With the increasing resolution of MRI, it has become possible to evaluate the pathophysiology of TOS in a minimally invasive, non-contrast-enhanced method, which was previously considered difficult. On the other hand, Furushima et al. reported that the maximum systolic blood flow velocity and the distance between the bases of the oblique muscle triangles on the first rib on ultrasonography reflected the severity of the disease [7]. Ultrasound is less invasive, but there are still problems in terms of procedural reproducibility and accuracy [24]. There are also cases of TOS with no obvious upper extremity or back pain, and they may not develop characteristic clinical symptoms [3,14].

A comprehensive diagnosis of TOS, which is a complex disease, requires a detailed medical history, assessment of clinical symptoms, and neurological examination. Hence, the presence of SCV disconnection on MRI-MIP images does not necessarily lead to the diagnosis of venous TOS, and there have been cases in which SCA stenosis was not observed on MRI-MIP images despite the presence of symptoms of SCA stenosis. The cases included in this study did not include “True TOS” [10,14], which is difficult to diagnose using MRI alone. However, disputed neurogenic TOS may be effective in excluding false positives. Apart from cases clearly attributable to sports, the absence of any vascular stenosis on MRI-MIP images in cases of suspected cervical spine origin or psychological cases is unlikely to indicate severe TOS requiring surgery. MRI-MIP images are useful in daily practice because they can be visually explained to the patients themselves. Moreover, when combined with sagittal and axial images taken simultaneously, as in Figure 4, it is possible to pick up the bone and soft tissue abnormalities and inflammation of the brachial plexus. However, if only MIP is used, only blood vessels can be detected.

In surgery, 3D-CT angiography is more useful than MRI because of its short imaging time to determine the position of the blood vessels in relation to the clavicle and ribs. However, it is difficult to perform this procedure in all patients with suspected TOS considering the use of contrast media and exposure to radiation. In this study, SCVs in surgical cases that were considered more severely stenosed showed a higher stenosis rate. Therefore, it is recommended that 3D-CT angiography be performed only in cases in which surgery is clinically indicated to avoid the unnecessary use of contrast media and exposure to radiation.

As to the cause of stenosis, the SCA, SCV, and nerve bundles are often stenosed even in the normal position, depending on the imaging position, as shown in previous studies in comparison to the normal side. In reviewing our surgical cases, most of the stenoses were caused by fibrous bands of the anterior scalene muscle and other anatomical factors [18,19]. In comparison with the healthy side, only the SCA in the surgery group tended to show strong stenosis, suggesting that anatomical factors of the anterior scalene muscle may be considered in cases in which there is a difference between the normal and affected sides of the SCA, which is less affected by posture. Moreover, the comparison with normal cases is warranted.

This study has two major limitations, which include the imaging of limb position and diagnosis of severity. The MRI imaging position was supine, and although the patient was in the Wright test position, there is a high likelihood that the SCA was not adequately compressed owing to high intravascular pressure and strong elasticity. The SCV also had a non-physiological compression and was not considered to fully reflect stenosis in the actual examination technique. Another problem with this method is that the patient must be in a symptomatic position for at least 20 min during imaging. Regarding TOS severity, in this study, surgical cases were compared with conservative treatment cases as severe. However, because surgery was decided solely based on the patient’s subjective assertion, it may not reflect severe organic stenosis, which may have caused variation in the data. Since the TOS itself lacks a clear quantitative index, further studies are needed to accumulate more cases in the future. In addition, other factors need to be considered for future study, which include cost-effectiveness, utility, indications for use, homogenization of sample size, and the ability to rule out other diagnoses.

## 5. Conclusions

On MRI-MIP images, the degree of stenosis of the SCV and SCA was classified into four grades, and the SCV was significantly more severely stenosed in the operated cases. Sagittal section images of the SCV, SCA, and nerve bundle showed significantly greater stenosis of the SCV in the operated cases but no significant difference in the SCA or nerve bundle between the operated and conservative treatment cases. MRI-MIP may be a useful adjunctive diagnostic method for understanding the stenosis status of vascular nerve bundles.

## Figures and Tables

**Table 1:** Background of patients in the surgery group.

	Age at Surgery	Affected Side	Cause or Occupation	Symptom	Duration of Illness (Years)	Classification of TOS	Co-Morbid Disease or Symptom	Satisfaction after Surgery
1	37	L	Baseball	Numbness after throwing	2	Disputed neurogenic		Good
2	15	R	Baseball	Numbness after throwing	2	Disputed neurogenic		Good
3	41	R	Baseball	Numbness after throwing	4	Disputed neurogenic		Good
4	17	R	Baseball	Numbness after throwing	2	Disputed neurogenic	UCL injury of same side elbow	Excellent
5	20	R	Baseball	Numbness after throwing	2	Disputed neurogenic		Good
6	19	R	Baseball	Numbness after throwing	3	Disputed neurogenic		Good
7	14	R	Tennis	Numbness and pain	2	Disputed neurogenic		Excellent
8	40	R	Reseaercher	Cold sensation and numbness	5	Arterial	Skin lesion of fingers, upper limb weakness	Excellent
9	43	L	Office worker	Cold sensation and numbness	3	Arterial	Numbness after jogging	Excellent
10	15	R	Malformation of first rib	Cold sensation and numbness	2	Arterial	Malformation of first rib	Excellent
11	56	L	Service industry	Cold sensation and numbness	6	Disputed neurogenic	Post operation of cervical stenosis	Good
12	32	L	Traffic accident	Numbness and pain	2	Disputed neurogenic		Fair
13	48	R	Service industry	Numbness and pain	6	Disputed neurogenic		Excellent
14	47	R	Nurse	Numbness and pain	5	Disputed neurogenic		Good
15	22	L	Office worker	Numbness and pain	3	Disputed neurogenic		Good
16	51	L	Care worker	Numbness and pain	15	Disputed neurogenic	Post operation of same side cubital tunnel syndrome	Good
17	26	L	Service industry	Numbness and pain	4	Disputed neurogenic		Good
18	24	R	Service industry	Numbness and pain	4	Disputed neurogenic		Excellent
19	55	L	Construction industry	Numbness and pain	4	Disputed neurogenic		Fair
20	53	L	Forestry industry	Numbness and pain	5	Disputed neurogenic		Good
21	44	L/R	Unemployed	Numbness and pain	10	Disputed neurogenic	Post operation of cervical stenosis	Good

TOS, thoracic outlet syndrome.

**Table 2:** Breakdown of SCV and SCA stenosis grade in MRI-MIP images.

		Grade 0	Grade 1	Grade 2	Grade 3	*p*-Value
SCV	Surgery (*n* = 22) Conservative (*n* = 91)	0 5	3 35	4 20	15 31	0.0113
SCA	Surgery (*n* = 22) Conservative (*n* = 91)	11 59	7 22	2 8	2 2	0.33

MRI-MIP, magnetic resonance imaging maximum-intensity protection; SCA, subclavian artery; SCV, subclavian vein.

**Table 3:** Stenosis rates of SCV, SCA, and nerve bundle.

	Surgery (%) *n* = 22	Conservative (%) *n* = 91	*p*-Value
SCV	76.7	67.7	0.036
SCA	34.6	28	0.21
Nerve bundle	34.5	34.5	0.53

SCA, subclavian artery; SCV, subclavian vein.

**Table 4:** Stricture rate of SCV, SCA, and nerve bundle on surgery and conservative group. There was a trend toward greater stenosis on the affected side of the SCA in the surgery group.

	Surgery (*n* = 7) Conservative (*n* = 24)	Affected Side	Normal Side	*p*-Value
SCV	Surgery Conservative	66.7 67.1	74.4 66.9	0.20 0.47
SCA	Surgery Conservative	47.4 29.3	28.5 32.5	0.064 0.28
Nerve bundle	Surgery Conservative	46.0 74.8	40.3 71.2	0.24 0.36

SCA, subclavian artery; SCV, subclavian vein.

## Data Availability

Not applicable.
